# Mn-induced Ferromagnetic Semiconducting Behavior with Linear Negative Magnetoresistance in Sr_4_(Ru_1−x_Mn_x_)_3_O_10_ Single Crystals

**DOI:** 10.1038/s41598-018-31679-w

**Published:** 2018-09-06

**Authors:** Lingyi Xing, Xin Gui, Weiwei Xie, Huibo Cao, Jiaqiang Yan, Brian C. Sales, Rongying Jin

**Affiliations:** 1 Department of Physics and Astronomy, Louisiana State University, Baton Rouge, LA 70803 USA; 2 Department of Chemistry, Louisiana State University, Baton Rouge, LA 70803 USA; 3 Neutron Scattering Division, Oak Ridge National Laboratory, Oak Ridge, TN 37831 USA; 4 Materials Science and Technology Division, Oak Ridge National Laboratory, Oak Ridge, TN 37831 USA

## Abstract

Triple-layered Sr_4_Ru_3_O_10_ is a unique ferromagnet with the central RuO_6_ layer behaving differently from two outer layers both crystallographically and magnetically. We report that the partial substitution of Ru by smaller Mn gives rise to modification in crystal structure, electronic and magnetic properties of Sr_4_(Ru_1−*x*_Mn_*x*_)_3_O_10_. Through the single crystal X-ray diffraction refinement, we find that (Ru/Mn)O_6_ octahedral rotation is no longer detectable at *x* ≥ 0.23, leading to the tetragonal structure. The magnetization measurements indicate the ferromagnetic transition temperature *T*_C_ decreases from 105 K for *x* = 0 to 30 K for *x* = 0.41, with the reduced magnetic moment as well. Remarkably, Mn doping results in the change of magnetic anisotropy from the easy *c* axis in *x* = 0 to the easy *ab* plane seen in *x* = 0.34 and 0.41. Such change also removes the *ab*-plane metamagnetic transition observed in *x* = 0. Furthermore, the electrical resistivity increases with increasing *x* showing semiconducting behavior with Δ ~ 10 meV for *x* = 0.34 and 30 meV for *x* = 0.41. Under applied magnetic field, the magnetoresistance exhibits negative and linear field dependence in all current and field configurations. These results clearly indicate Sr_4_(Ru_1−x_Mn_x_)_3_O_10_ is a novel ferromagnetic semiconductor with exotic magnetotransport properties.

## Introduction

The Ruddlesden-Popper (RP) ruthenates Sr_*n*+1_Ru_*n*_O_3*n*+1_ (*n* = 1, 2, 3 … ∞) have attracted great attention because of their exotic electronic and magnetic properties. While the only difference in their chemical composition is the number of RuO_6_ octahedral layers *n* in the unit cell, their physical properties vary from a *p*-wave superconductor (*n* = 1)^1^, to a paramagnetic metal with magnetic field-induced quantum critical point (*n* = 2)^2^, to an unusual ferromagnetic metal with in-plane metamagnetism (*n* = 3)^3^, to a ferromagnetic polar metal (*n* = ∞)^4^. Both experimental^3,5–10^ and theoretical studies^10–12^ indicate that the fundamental building block RuO_6_ octahedron plays an extremely important role in these unconventional properties. In the RP series, RuO_6_ octahedra are connected by corner sharing O atoms within the *n* layers, which can be distorted in multiple ways such as elongation, compression, rotation, and tilt. For example, with increasing *n*, the rotation angle of RuO_6_ octahedron changes from zero (*n* = 1)^13^, to 7° (*n* = 2)^14,15^, to 11.2° (*n* = 3)^16^ to ~12° (*n* = ∞). According to theoretical calculations^11,17,18^, such distortion impacts the electronic distribution, thus changing the physical properties.

In the RP series, the members with odd *n* are particularly interesting. For Sr_2_RuO_4_ with *n* = 1, the RuO_6_ octahedron rotates about 9° at the surface^10^, even it is absent in bulk. Such rotation may stabilize ferromagnetism at the surface, which is ultimately connected to the Cooper pair formation with parallel spins in bulk. For Sr_4_Ru_3_O_10_ with *n* = 3, the RuO_6_ octahedron in the central layer apparently rotates double amount (~11.2°) than that in the two outer layers (~5.6°), corresponding to different magnetic moment as well^16^. According to density functional calculations^11,12^, the orthorhombic structure with the rotation of the RuO_6_ octahedron is in favor of ferromagnetic coupling. In the previous study of Sr_3_(Ru_1−x_Mn_x_)_2_O_7_, partial replacement of Ru by smaller Mn leads to the decrease of (Ru/Mn)O_6_ octahedral rotation, and long-range antiferromagnetic (AFM) ordering at T_N_^8^. Intriguingly, T_N_ versus *x* is dome-like with both T_N_ and the rotation vanishes at the same doping level. This suggests that the correlation between octahedral rotation and magnetic coupling is more complex than simple monotonic response. Given its magnetic ground state and the difference in local structure between outer and central layers^19,20^, Sr_4_Ru_3_O_10_ could be much more susceptible to Mn doping than the case of n = 2. In this article, we report, for the first time, the experimental investigation of Mn doped Sr_4_Ru_3_O_10_ single crystals, including the crystal structure, magnetization, and electrical transport properties. The partial replacement of Ru by Mn results in the modification of (1) crystal structure by removing octahedral rotation, (2) magnetic interaction by changing the easy axis from the *c* direction to the *ab* plane, and (3) electrical conduction from metallic to semiconducting with unusual magnetotransport behavior.

## Results and Discussion

For both nominal *x* = 0.25 and 0.5 crystals, our single-crystal X-ray diffraction refinement results in structural and compositional information that are given in Tables 1 and 2, respectively. The refinement indicates that they form a tetragonal structure (S.G. I4/mmm) with actual *x* = 0.23 and 0.41, respectively. Note that the actual Mn concentration is the average value of Mn in the central layer (Mn1) and outer layers (Mn2). While Mn is randomly distributed within each layer, our structural refinement indicates that Mn1 concentration is slightly lower than Mn2 in both *x* = 0.23 and 0.41. As summarized in Tables 1 and 2 for *x* = 0.23 and 0.41, the atomic coordination and occupancy correspond to the Sr_4_(Ru_1−x_Mn_x_)_3_O_10_ phase with lattice parameters *a* = *b* = 3.9033(5) Å and *c* = 28.138(3) Å for *x* = 0.23, and *a* = *b* = 3.910(4) Å and *c* = 27.96(3) Å for *x* = 0.41. The doping concentration *x* obtained from XRD is consistent with the element analysis through wavelength dispersive spectroscopy (WDS) via Joel JXA-8230 Electron Microprobe. The WDS measurements of nominal *x* = 0.4 single crystals give actual *x* = 0.34, which are used for the investigations of electrical and magnetic properties presented below.

**Table 1:** Atomic coordinates and equivalent isotropic displacement parameters of Sr_4_(Ru_0.77(1)_Mn_0.23_)_3_O_10_ at 300 (2) K obtained through single crystal X-ray diffraction refinement.

Atom	Wyckoff.	Occupancy.	*x*	*y*	*z*	*U*_*eq*_
Sr1	4*e*	1	0	0	0.43011 (4)	0.0092 (3)
Sr2	4*e*	1	0	0	0.29737 (4)	0.0074 (3)
(Ru/Mn) 1	4*e*	0.784 (8)/0.216	½	½	0.35987 (3)	0.0045 (3)
(Ru/Mn) 2	2*a*	0.741 (8)/0.259	½	½	½	0.0033 (4)
O1	4*e*	1	½	½	0.2896 (2)	0.009 (2)
O2	4*e*	1	½	½	0.4310 (4)	0.016 (2)
O3	8*g*	1	½	0	0.3610 (2)	0.014 (1)
O4	4*c*	1	½	0	½	0.043 (4)

U_*eq*_ is defined as one-third of the trace of the orthogonalized U_*ij*_ tensor (Å^2^).

**Table 2:** Atomic coordinates and equivalent isotropic displacement parameters of Sr_4_(Ru_0.59(1)_Mn_0.41_)_3_O_10_ at 300(2)K obtained through single crystal X-ray diffraction refinement.

Atom	Wyckoff.	Occupancy.	*x*	*y*	*z*	*U*_*eq*_
Sr1	4*e*	1	0	0	0.43022 (4)	0.0093 (3)
Sr2	4*e*	1	0	0	0.29779 (3)	0.0076 (3)
(Ru/Mn)1	4*e*	0.596(8)/0.404	½	½	0.35989 (3)	0.0045 (3)
(Ru/Mn)2	2*a*	0.575(8)/0.425	½	½	½	0.0034 (4)
O1	4*e*	1	½	½	0.2906 (2)	0.010 (2)
O2	4*e*	1	½	½	0.4309 (2)	0.014 (2)
O3	8*g*	1	½	0	0.3609 (2)	0.012 (1)
O4	4*c*	1	½	0	½	0.038 (3)

U_*eq*_ is defined as one-third of the trace of the orthogonalized U_*ij*_ tensor (Å^2^).

**Figure 1:**
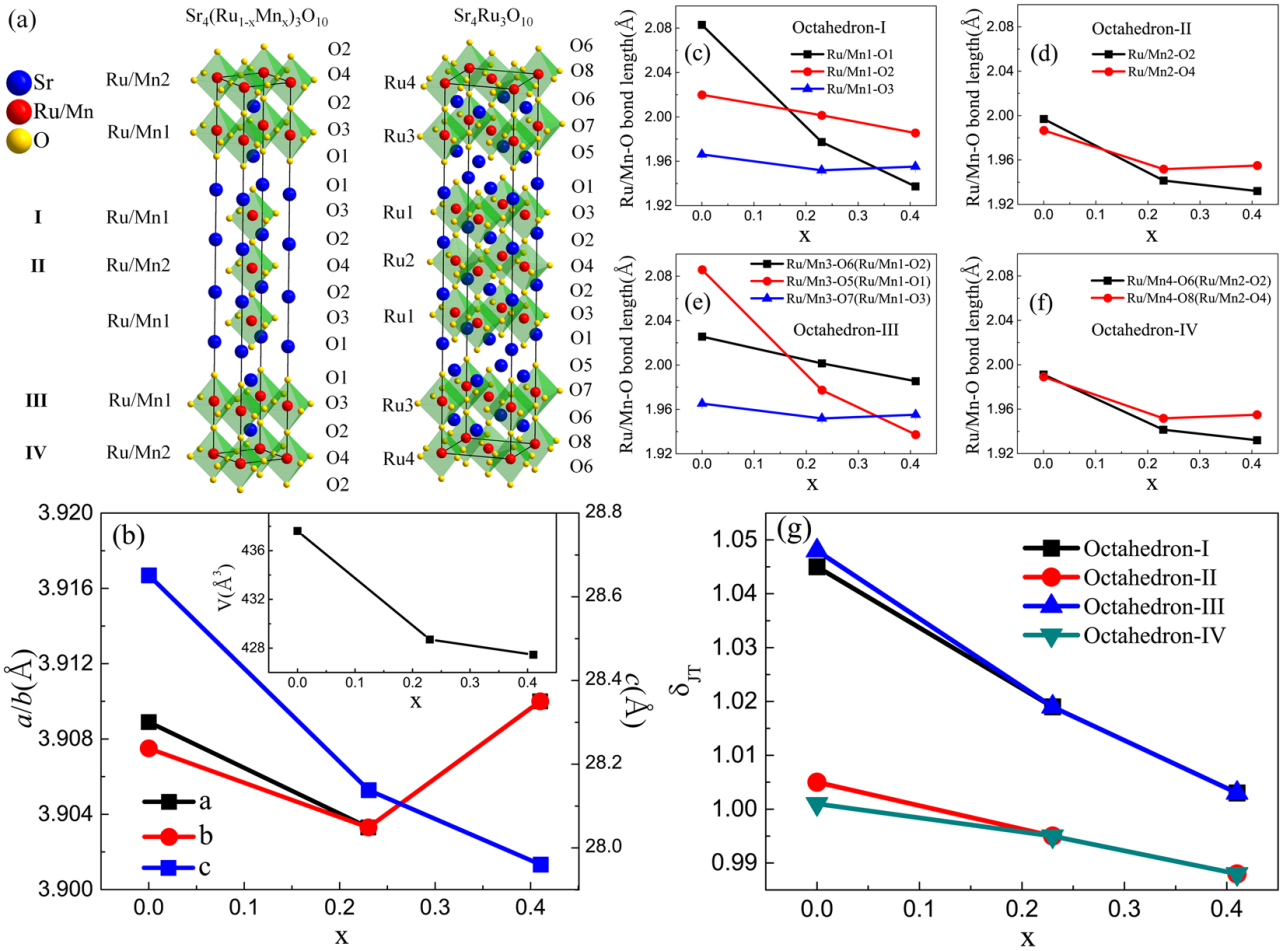
(**a**) Crystal structure of Sr_4_(Ru_1−x_Mn_x_)_3_O_10_ (*x* = 0.23 and 0.41) (left) and the parent compound Sr_4_Ru_3_O_10_ (right)^3^. (**b**) Doping (*x*) dependence of lattice parameters *a*, *b*, and *c*. For *x* = 0, *a*/√2 and *b*/√2 are used. The inset is the *x* dependence of the unit cell volume *V*. (**c**–**f**) Doping (*x*) dependence of the Bond lengths of (Ru/Mn)-O in four different octahedral. (**g**) Doping (*x*) dependence of the Jahn-Teller distortion δ_JT_ for four different octahedra.

Figure 1(a) depicts the crystal structure of Sr_4_(Ru_1−x_Mn_x_)_3_O_10_ for *x* = 0.23 and 0.41, with the indication of two Ru/Mn ((Ru/Mn)1 and (Ru/Mn)2) sites and four O (O1, O2, O3, O4) sites. Compared to the undoped case (*x* = 0) as shown in the right of Fig. 1(a), both the number of Ru/Mn and O sites are reduced. The change from orthorhombic for x = 0 to tetragonal for *x* = 0.23, and 0.41 indicates the removal of octahedral rotation upon Mn doping. While *a* (*b*) (the values for parent compound are divided by $\sqrt{2}$) remains more or less a constant, lattice parameter *c* decreases with increasing *x* as plotted in Fig. 1(b). This means that the unit cell volume decreases with increasing *x* (see the inset of Fig. 1(b)). To understand the origin of unit cell shrinkage, we plot the *x* dependence of the bond lengths of Ru/Mn-O in four different octahedra (I, II, III and IV, referred from *x* = 0) in Fig. 1(c–f), respectively. With increasing *x*, while the in-plane bond length slightly decreases, the out-of-plane Ru/Mn-O bond length decreases more dramatically. By calculating the ratio of the average out-of-plane to in-plane Ru/Mn-O distance, we obtain the *x* dependence of the Jahn-Teller (JT) distortion δ_JT_ for I, II, III and IV, which are shown in Fig. 1(g). Note that δ_JT_ for outer-layer octahedra (I and III) decrease from 1.05 to 1.00 while those for central-layer octahedra (II and IV) decrease from 1.01 to 0.99, as *x* increases from 0 to 0.41. These indicate Mn doping makes the outer-layer octahedra less elongated, and the central-layer ones slightly compressed.

**Figure 2:**
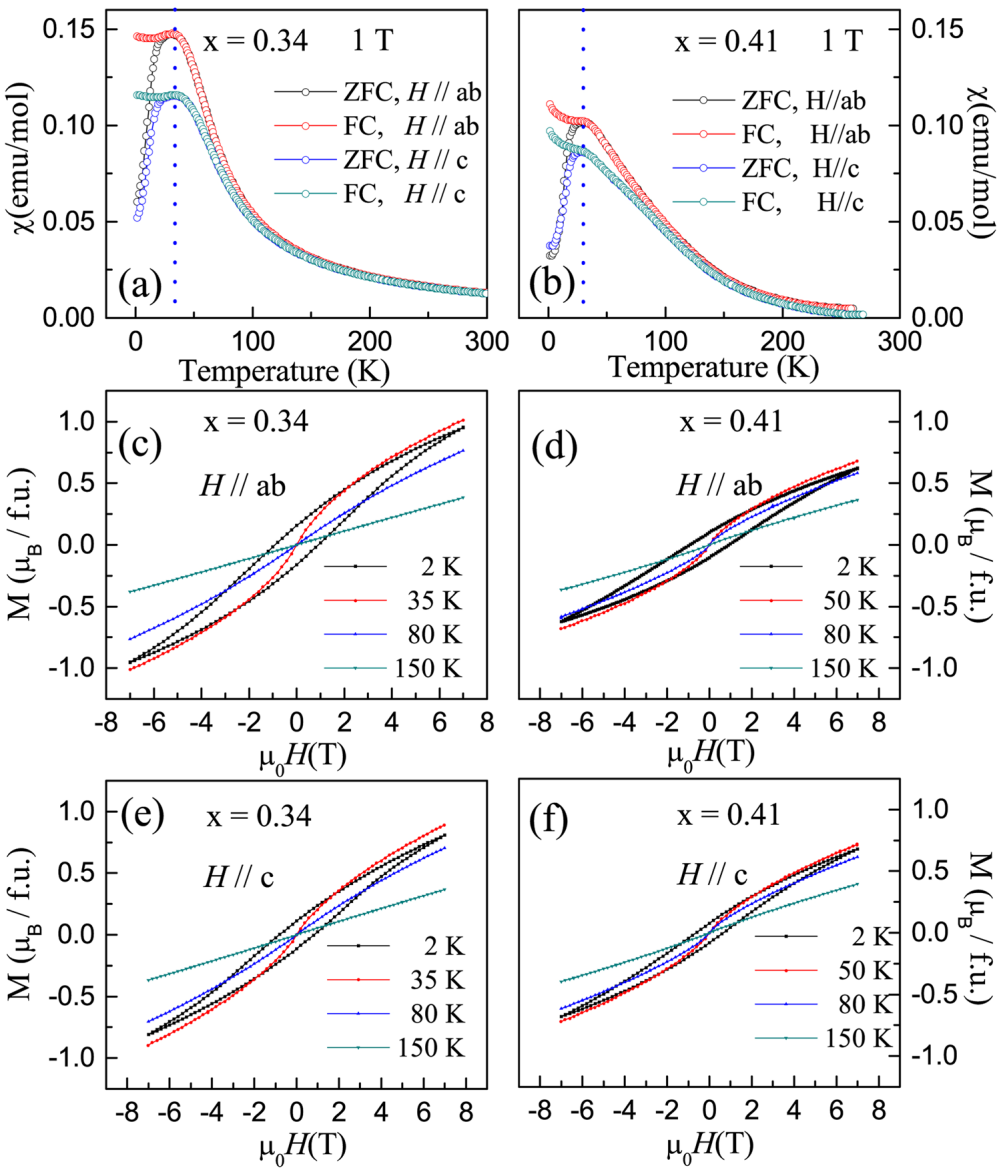
(**a**,**b**) Temperature dependence of the magnetic susceptibility χ for *x* = 0.34 and 0.41 single crystals measured by applying *H* = 1 T alongThe Ruddlesden-Popper (RP) ruthenates the *ab* plane and *c* axis, respectively. The blue dotted lines represent the ferromagnetic transition temperature. (**c**,**d**) Magnetization hysteresis loops at different temperatures for *x* = 0.34 and 0.41 single crystals when *H*//*ab*. (**e**,**f**) Magnetization hysteresis loops at different temperatures for *x* = 0.34 and 0.41 single crystals when *H*//*c*.

Figure 2(a,b) show the temperature dependence of the magnetic susceptibility for *x* = 0.34 and 0.41 measured with *H* ( = 1 T) parallel to the *ab* plane (χ_ab_) and *c* axis (χ_c_), respectively. While the overall profile is similar to that observed in *x* = 0^3^, several features are worth noting: (1) both χ_ab_ and χ_c_ only reflect one magnetic transition *T*_C_, which is 35 K for *x* = 0.34 and 30 K for *x* = 0.41; (2) χ_ab_ > χ_c_ for *x* = 0.34 and 0.41, indicating that the magnetic easy axis is along the *ab* plane. Compared to the case of *x* = 0, *T*_C_ obviously is decreased and the magnetic easy axis is switched from the *c* direction to the *ab* plane upon Mn doping. In analyzing the high temperature susceptibility data using the Curie–Weiss law χ = χ_0_ + $\frac{{N}_{A}{\mu }_{{\rm{eff}}}^{2}\,/\,3{k}_{B}\,}{T-{\theta }_{CW}}$ (χ_0_ is a constant, *N*_A_ is the Avogadro constant, and *k*_B_ the Boltzmann constant), we obtain positive Curie-Weiss temperature *θ*_CW_ and effective moment μ_eff_. For *x* = 0.34, χ_0_ ~ −0.003 emu/mol, *θ*_CW_ ~ 33 K and μ_eff_ ~ 3.4μ_B_, and χ_0_ ~ −0.01 emu/mol, *θ*_CW_ ~ 37 K and μ_eff_ ~ 3.2μ_B_ for *x* = 0.41. The positive *θ*_CW_ value indicates that magnetic interaction is ferromagnetic along both the *ab* plane and *c* axis, similar to the *x* = 0 case^3^. However, for *x* = 0.41, θ_CW_ is higher than *T*_C_, which usually occurs in low-dimensional or frustrated magnetic systems. While there is little anisotropy in magnetic susceptibility above *θ*_CW_, the tetragonal structure disfavors magnetic frustration as well. One possibility is that the compressed central (Ru/Mn)O_6_ layer [see Fig. 1(g)] may be in favor of antiferromagnetic interaction, while the elongated two outer (Ru/Mn)O_6_ layers support ferromagnetic interaction according to theoretical calculations^11,12^. The mixed ferromagnetic and antiferromagnetic interactions result in higher *θ*_CW_ but smaller μ_eff_ in *x* = 0.41 compared to the case of *x* = 0.34. Although similar argument also should apply to the latter case, antiferromagnetic interaction is less dramatic own to smaller or close-to-zero compression of the central (Ru/Mn)O_6_ octahedra [see Fig. 1(g)]. Nevertheless, the estimated μ_eff_ for *x* = 0.34, and 0.41 corresponds to effective spin 1 < *S*_eff_ ~1.19–1.28 < 3/2 for Ru/Mn, slightly higher than the undoped case with *S* = 1^21^. This suggests that Mn has the same valence as Ru^4+^, i.e., Mn^4+^, which gives *S* = 3/2.

To further identify the nature of the magnetic interaction in the doped systems, the magnetization hysteresis is measured at various temperatures, which is presented in Fig. 2(c–f) for both *x* = 0.34 and 0.41 with *H*//*ab* and *H*//*c*, respectively. In both directions, *M*(*H*) is linear at high temperatures for *x* = 0.34 and 0.41. As temperature is lowered, non-linear *M*(*H*) is observed at low fields as seen at *T* = 80 K. However, the hysteresis loop does not occur until *T* approaches *T*_C_, consistent with long-range ferromagnetic ordering. More importantly, there is no sudden increase upon increasing magnetic field in either *M*_ab_ or *M*_c_. This indicates the absence of the metamagnetic transition up to 7 Tesla in both *x* = 0.34 and 0.41. Furthermore, the magnetic moment at 7 T decreases with increasing Mn doping level, consistent with the scenario that Mn doping increases antiferromagnetic interaction.

**Figure 3:**
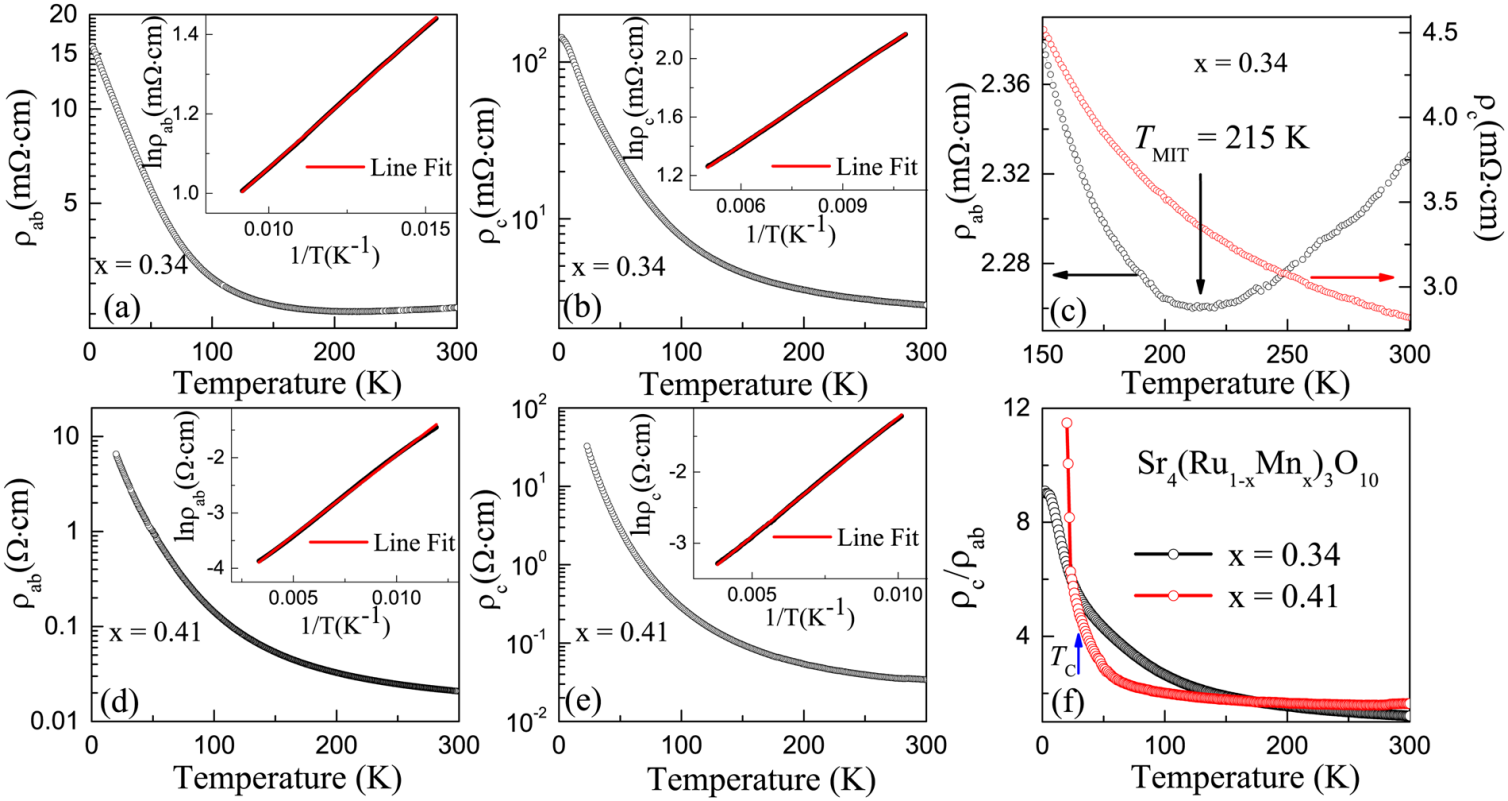
The temperature dependence of (**a**) the *ab*-plane resistivity ρ_ab_ and (**b**) the *c*-axis resistivity ρ_c_, and (**c**) the enlarged ρ_ab_ and ρ_c_ for *x* = 0.34 single crystal. The temperature dependence of (**d**) the *ab*-plane resistivity ρ_ab_ and (**e**) the *c*-axis resistivity ρ_c_ for *x* = 0.41 single crystal, and (**f**) the anisotropy ρ_c_/ρ_ab_. The insets for both (**a**,**b**,**d**) and (**e**) show the replot of ρ_ab_(T) and ρ_c_(T), and fitting curves (red) according to the thermal activation model ρ = ρ_0_exp(Δ/*κ*_B_T).

According to previous studies, small percentage of Mn doping in single-layered Sr_2_RuO_4_ (n = 1)^22^ and double-layered Sr_3_Ru_2_O_7_ (n = 2)^8,23^ results in antiferromagnetic insulating ground state. The same trend is observed in SrRuO_3_ (n = ∞), in which more than 39% Mn doping turns the system into an AFM insulator as well^24,25^. The fact that Sr_4_(Ru_1−x_Mn_x_)_3_O_10_ (*x* = 0.34 and 0.41) retains its ferromagnetic (FM) ordering is remarkable, suggesting that the magnetic interaction in the n = 3 system is different from those mentioned above. Figure 3(a–e) show temperature dependence of both in-plane (ρ_ab_) and c-axis (ρ_c_) resistivities for *x* = 0.34 (a–c) and 0.41 (d–e), respectively. Note that, for *x* = 0.34, ρ_c_ shows a semiconducting behavior in the entire measured temperature range while the metal-insulator transition (MIT) temperature occurs at 215 K in the *ab*-plane (see Fig. 3(c)). For *x* = 0.41, we can speculate that this transition is beyond 300 K, as both ρ_ab_ and ρ_c_ are non-metallic below 300 K. While no anomaly is observed in ρ_ab_ and ρ_c_ at *T*_C_, the resistivity anisotropy ρ_c_/ρ_ab_, presented in Fig. 3(f), shows steep change below *T*_C_ than that at higher temperatures. This is consistent with the magnetic anisotropy that stronger in-plane ferromagnetism (*M*_ab_ > *M*_c_) results in better electrical conduction along the *ab* plane. By replotting the temperature dependence of resistivities as lnρ versus T^−1^ in the inset of Fig. 3(a,b,d and e), liner relationship is clearly revealed. This indicates that both ρ_ab_(T) and ρ_c_(T) can be described by the thermal activation model ρ = ρ_0_exp(Δ/*k*_B_T), where ρ_0_ is a temperature-independent constant, and Δ is the thermal activation energy. By fitting experimental data to the formula, we obtain Δ ~10 meV and 30 meV for *x* = 0.34 and 0.41, respectively. The solid lines in the insets of Fig. 3(a,b,d and e) represent the fitting results, which describe the experimental data very well.

**Figure 4:**
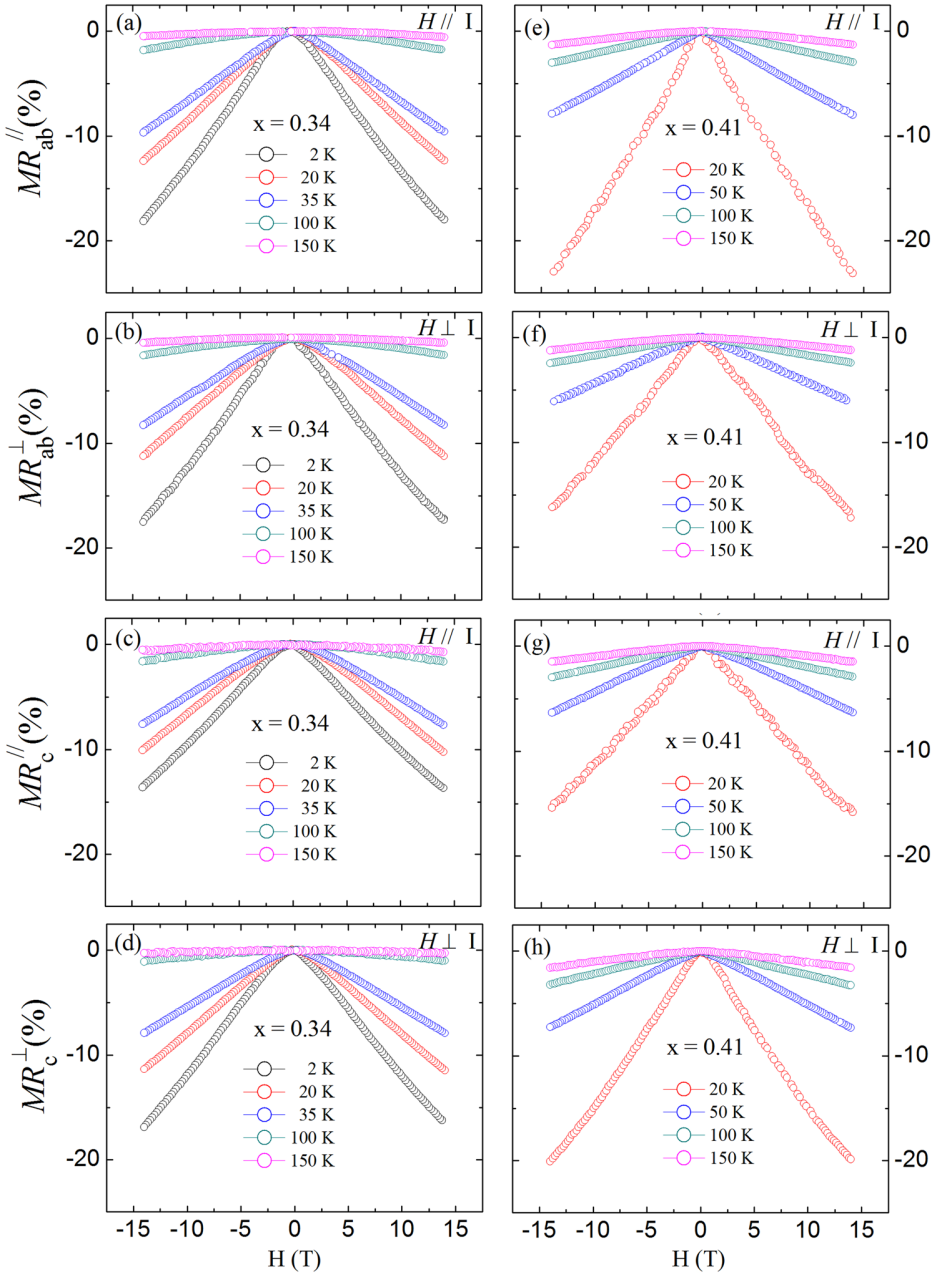
Magnetic field dependence of MR for *x* = 0.34 (**a**–**d**) and 0.41 (**e**–**h**) single crystals measured in the configurations of *I*//*ab* and *H*//*I* (**a**,**e**), *I*//*ab* and *H* ⊥ *I* (**b**,**f**), *I*//*c* and *H*//*I* (**c**,**g**), and *I*//*c* and *H* ⊥ *I* (**d**,**h**).

The above electrical and magnetic properties indicate that the Mn-doped Sr_4_(Ru_1−x_Mn_x_)_3_O_4_ (*x* = 0.34 and 0.41) is a *ferromagnetic semiconductor with a narrow energy gap*. This sets it apart from other sister compounds with Mn doping and adds a new member in a small magnetic semiconductor family. Further evidence for ferromagnetic semiconducting properties of Sr_4_(Ru_1−x_Mn_x_)_3_O_4_ (*x* = 0.34 and 0.41) is reflected in magnetoresistance (MR). Figure 4(a–d) show, for *x* = 0.34, the field dependence of the MR for *I*//*ab* and *H*//*I* ($M{R}_{ab}^{//}$), *I*//*ab* and *H* ⊥ *ab* ($M{R}_{ab}^{\perp }$), *I*//*c* and *H*//*c* ($M{R}_{c}^{//}$), and *I*//*c* and *H* ⊥ *c* ($M{R}_{c}^{\perp }$), respectively. Several features are worth noting. First, the MR in all field and current configurations is *negative* at T ≤ 150 K. Second, $|M{R}_{ab}^{//}|$ > $|M{R}_{ab}^{\perp }|$ and $|M{R}_{c}^{\perp }|$ > $\,|M{R}_{c}^{//}|$. Third, MR measured in all configurations exhibits more or less linear field dependence without the sign of saturation up to 14 Tesla. The same features also are observed for *x* = 0.41 as shown in Fig. 4(e–h).

The negative MR for Sr_4_(Ru_1−x_Mn_x_)_3_O_4_ (*x* = 0.34 and 0.41) is different from that seen in the parent compound, which is positive along both the *ab* plane and *c* direction prior to the metamagnetic transition field^26,27^. This confirms that the magnetic properties of Mn-doped system are different from the undoped case. Furthermore, the fact that $|M{R}_{ab}^{//}|$ > $|M{R}_{ab}^{\perp }|$ and $|M{R}_{c}^{\perp }|$>$\,|M{R}_{c}^{//}|$ indicate that spin scattering is strong in the *ab* plane, and can be more effectively suppressed when *H*//*ab*. This is consistent with the observation that the magnetic easy axis in Sr_4_(Ru_1−x_Mn_x_)_3_O_4_ (*x* = 0.34 and 0.41) is along the *ab* plane. The most remarkable feature is the linear-*H* dependence of negative MR in all configurations. While it is discussed in both classic and quantum scenarios, linear negative MR (LNMR) in all configurations is rare.

In normal circumstances, one would expect that MR exhibits the *H*^2^ dependence in low fields and eventually saturates at high fields^28^. Linear MR can be found in materials with open Fermi surfaces^29^, disordered metals or semiconductors^30–33^, and in the extreme quantum limit with $\hslash {\omega }_{c} > {E}_{F}$ (where *ω*_*c*_ is the cyclotron frequency and E_F_ is the Fermi energy)^34–36^. However, the MR is usually positive in these scenarios. LNMR has been discussed in non-magnetic disordered 2D gas systems with random corrugation and defects^37–41^, and polycrystalline graphite consisting of nano-sized particles^42^. In the latter case, the system exhibits semiconducting behavior with thermally activated electrical conduction. Due to its polycrystalline nature, it consists of weak localization (WL)^43^ and diffuse scattering (DS) between grain boundaries^44^, giving rise to MR_total_ = MR_WL_ + MR_DS_ = −K_1_H^1/2^ − K_2_H^2^ (K_1_ and K_2_ are constants)^42^. Experimentally, one would observe LNMR as the consequence of these two contributions. As T approaches 0 K, MR_WL_ becomes dominant, leading to cusp-shaped H dependence of MR. While Sr_4_(Ru_1−x_Mn_x_)_3_O_10_ exhibits thermally activated semiconducting conduction (see Fig. 3), the above scenario may not be feasible to explain LNMR in our case, because (1) our samples are single crystals with little or no grain boundaries and (2) there is no sign for cusp-shaped H dependence in MR up to 14 Tesla down to 2 K (see Fig. 4).

For Sr_4_(Ru_1−x_Mn_x_)_3_O_10_, the observed LNMR should be related to Mn doping as it is (1) absent in *x* = 0^26,27^, (2) larger in higher Mn doping level under the same condition (see Fig. 4), and (3) appearing below and above *T*_C_. The fact that LNMR occurs in all current and field configurations with small anisotropy for both *x* = 0.34 and 0.41 indicates that it is not related to dimensional confinement nor orbital nature in our system. Similar behavior was observed in Be-doped AlGaAs – GaAs quantum well structures, in which the LNMR is attributed to spin exchange interactions between localized states^40^. Under the application of magnetic field, spin disorder related to exchange interactions is suppressed and Zeeman energy activates carriers in the localized states to delocalized states, leading to LNMR^40,45^. This is consistent with our observation that the largest LNMR occurs when H is applied along the magnetic easy (*ab*-) plane and continuous increase of magnetization (see Fig. 2). However, this scenario cannot explain the increased LNMR with increasing *x*, as energy gap is enlarged and spin polarization becomes weaker. Theoretically, MR in doped ferromagnetic semiconductors is expected to be inversely proportional to the number of charge carriers per magnetic unit cell^30^. Our data is consistent with this picture, as Mn doping results in charge localization thus reducing charge carrier density in Sr_4_(Ru_1−x_Mn_x_)_3_O_10_.

In summary, we have successfully grown Mn-doped single crystalline Sr_4_(Ru_1−x_Mn_x_)_3_O_10_ with *x* up to 0.41. Single crystal X-ray diffraction indicates that the partial substitution of Ru by Mn results in symmetry change from orthorhombic to tetragonal due to the removal of octahedral rotation. Both electrical and magnetic properties are changed as well. For *x* = 0.34 and 0.41, the system becomes ferromagnetic semiconducting with *T*_C_ ~35 K and energy gap ~10 meV for *x* = 0.34 and *T*_C_ ~30 K and 30 meV for *x* = 0.41, making Sr_4_(Ru_1−x_Mn_x_)_3_O_10_ an excellent material system to study ferromagnetic semiconducting properties. One of remarkable features is its linear negative MR observed in a wide temperature and field range in all current and field configurations. Such behavior may be explained by considering spin exchange interactions between localized states, which likely increases with increasing Mn doping. Our results shed important insights for linear MR particularly in the case of LNMR.

## Methods

High-quality Sr_4_(Ru_1−x_Mn_x_)_3_O_10_ single crystals were grown via the floating-zone technique. For making both feed and seed rods, polycrystalline Sr_4_(Ru_1−x_Mn_x_)_3_O_10_ was first prepared by heating the mixture with the molar ratio SrCO_3_: [(1 − *x*)Ru + *x*MnO_2_] = 4: 3 up to 1400 °C for 24 h in oxygen atmosphere with quick cool off by quenching. It is then hydrostatically pressed into cylinder-shaped rods, and further sintered at 950 °C for another 12 h in oxygen atmosphere. Single crystals were grown through melting the feed rod and moving downward with the speed of 30 mm/h. To prevent oxygen deficiency, 0.9 MPa oxygen pressure was applied during the growth. In addition, both feed and seed rods were rotated in opposite directions at 20 rpm to improve homogeneity and reduce possible impurity.

For single-crystal X-ray diffraction (XRD), a single crystal was mounted on the tip of Kapton loop, and data were collected on a Bruker Apex II X-ray diffractometer with Mo radiation K α_1_ (λ = 0.71073 Å). The SMART software was used for data acquisition over a full sphere of reciprocal space with 0.5° scans in ω with an exposure time of 10 s per frame. The 2θ range extended from 0° to 80°. Intensities were extracted and corrected for Lorentz and polarization effects with the SAINT program. Numerical absorption corrections were accomplished with XPREP which is based on face-indexed absorption. The crystal structure was determined based on full-matrix least-squares methods using the SHELXTL package^46^.

Physical property measurements were investigated in *x* = 0.34 and 0.41 single crystals. The magnetization was measured using the magnetic property measurement system (MPMS, *Quantum Design*), while the electrical resistivity was carried out in the physical property measurement system (PPMS, *Quantum Design*).

## References

[1] Maeno Y (1994). Superconductivity in a layered perovskite without copper. Nature.

[2] Grigera SA (2001). Magnetic Field-Tuned Quantum Criticality in the Metallic Ruthenate Sr_3_Ru_2_O_7_. Science.

[3] Crawford MK (2002). Structure and magnetism of single crystal Sr_4_Ru_3_O_10_: A ferromagnetic triple-layer ruthenate. Phys. Rev. B.

[4] Callaghan A, Moeller CW, Ward R (1966). Magnetic interactions in ternary ruthenium oxides. Inorg Chem.

[5] Friedt O (2001). Structural and magnetic aspects of the metal-insulator transition in Ca_2−x_Sr_x_RuO_4_. Phys. Rev. B.

[6] Ikeda S-I, Maeno Y, Nakatsuji S, Kosaka M, Uwatoko Y (2000). Ground state in Sr_3_Ru_2_O_7_: Fermi liquid close to a ferromagnetic instability. Phys. Rev. B.

[7] Perry RS (2001). Metamagnetism and Critical Fluctuations in High Quality Single Crystals of the Bilayer Ruthenate Sr_3_Ru_2_O_7_. Phys. Rev. Lett..

[8] Hu B (2011). Structure-property coupling in Sr_3_(Ru_1−x_Mn_x_)_2_O_7_. Phys. Rev. B.

[9] Chen C (2016). Hidden phases revealed at the surface of double-layered Sr_3_(Ru_1−x_Mn_x_)_2_O_7_. Phys. Rev. B.

[10] Matzdorf R (2000). Ferromagnetism Stabilized by Lattice Distortion at the Surface of the p-Wave Superconductor Sr_2_RuO_4_. Science.

[11] Fang Z, Terakura K (2001). Magnetic phase diagram of Ca_2-x_Sr_x_RuO_4_ governed by structural distortions. Phys. Rev. B.

[12] Singh DJ, Mazin II (2001). Electronic structure and magnetism of Sr_3_Ru_2_O_7_. Phys. Rev. B.

[13] Huang Q (1994). Neutron Powder Diffraction Study of the Crystal Structures of Sr_2_RuO_4_ and Sr_2_IrO_4_ at Room Temperature and at 10 K. J Solid State Chem.

[14] Huang Q, Lynn JW, Erwin RW, Jarupatrakorn J, Cava RJ (1998). Oxygen displacements and search for magnetic order in Sr_3_Ru_2_O_7_. Phys. Rev. B.

[15] Shaked H, Jorgensen JD, Chmaissem O, Ikeda S, Maeno Y (2000). Neutron Diffraction Study of the Structural Distortions in Sr_3_Ru_2_O_7_. J Solid State Chem.

[16] Weickert F (2017). Missing magnetism in Sr_4_Ru_3_O_10_: Indication for Antisymmetric Exchange Interaction. Scientific Reports.

[17] Malvestuto M (2011). Electronic structure trends in the Sr_n+1_Ru_n_O_3n+1_ family (n = 1,2,3). Phys. Rev. B.

[18] Malvestuto M (2013). Nature of the apical and planar oxygen bonds in the Sr_n+1_Ru_n_O_3n+1_ family (n = 1,2,3). Phys. Rev. B.

[19] Carleschi E (2014). Double metamagnetic transition in Sr_4_Ru_3_O_10_. Phys. Rev. B.

[20] Weickert F (2018). In-depth study of the H−T phase diagram of Sr_4_Ru_3_O_10_ by magnetization experiments. Physica B.

[21] Cao G, McCall SK, Crow JE, Guertin RP (1997). Ferromagnetism in Sr_4_Ru_3_O_10_: Relationship to other layered metallic oxides. Phys. Rev. B.

[22] Ortmann JE (2013). Competition Between Antiferromagnetism and Ferromagnetism in Sr_2_RuO_4_ Probed by Mn and Co Doping. Scientific Reports.

[23] Mathieu R (2005). Impurity-induced transition to a Mott insulator in Sr_3_Ru_2_O_7_. Phys. Rev. B.

[24] Cao G (2005). Itinerant ferromagnetism to insulating antiferromagnetism: A magnetic and transport study of single crystal SrRu_1−x_Mn_x_O_3_(0 ≤ × < 0.60). Phys. Rev. B.

[25] Horiba K (2010). Electronic structure of SrRu_1−x_Mn_x_O_3_ studied by photoemission and x-ray absorption spectroscopy. Phys. Rev. B.

[26] Fobes D (2007). Phase diagram of the electronic states of trilayered ruthenate Sr_4_Ru_3_O_10_. Phys. Rev. B.

[27] Fobes D (2010). Anisotropy of magnetoresistivities in Sr_4_Ru_3_O_10_: Evidence for an orbital-selective metamagnetic transition. Phys. Rev. B.

[28] Pippard, A. B. Magnetoresistance in metals. *Cambridge University Press*, Cambridge (1989).

[29] Kapitza P (1929). The change of electrical conductivity in strong magnetic fields. Proceedings of the Royal Society of London A.

[30] Majumdar P, Littlewood PB (1998). Dependence of magnetoresistivity on charge-carrier density in metallic ferromagnets and doped magnetic semiconductors. Nature.

[31] Parish MM, Littlewood PB (2003). Non-saturating magnetoresistance in heavily disordered semiconductors. Nature.

[32] Parish MM, Littlewood PB (2005). Classical magnetotransport of inhomogeneous conductors. Phys. Rev. B.

[33] Pan J, Karki A, Plummer EW, Jin R (2017). Doping effect on the physical properties of Ca_10_Pt_3_As_8_(Fe_2_As_2_)_5_ single crystals. Journal of Physics: Condensed Matter.

[34] Abrikosov AA (1998). Quantum magnetoresistance. Phys. Rev. B.

[35] Abrikosov AA (2000). Quantum linear magnetoresistance. Europhys Lett.

[36] Abrikosov AA (2003). Quantum linear magnetoresistance; solution of an old mystery. Journal of Physics A: Mathematical and General.

[37] Dmitriev A, Dyakonov M, Jullien R (2002). Anomalous Low-Field Classical Magnetoresistance in Two Dimensions. Phys. Rev. Lett..

[38] Cheianov VV, Dmitriev AP, Kachorovskii VY (2003). Anomalous negative magnetoresistance caused by non-Markovian effects. Phys. Rev. B.

[39] Sotomayor NM (2004). Negative linear classical magnetoresistance in a corrugated two-dimensional electron gas. Phys. Rev. B.

[40] Agrinskaya NV, Kozub VI, Mikhailin NY, Shamshur DV (2017). Spin-controlled negative magnetoresistance resulting from exchange interactions. JETP Lett..

[41] Schluck J (2018). Linear negative magnetoresistance in two-dimensional Lorentz gases. Phys. Rev. B.

[42] Zhang X, Xue QZ, Zhu DD (2004). Positive and negative linear magnetoresistance of graphite. Phys Lett A.

[43] Hishiyama Y, Irumano H, Kaburagi Y, Soneda Y (2001). Structure, Raman scattering, and transport properties of boron-doped graphite. Phys. Rev. B.

[44] Fujita S (1968). Negative magnetoresistance in carbons and diffuse scattering at crystallite boundaries. Carbon.

[45] Fukuyama, H. & Yoshida, K. Negative Magnetoresistance in the Anderson Localized States. *J.Phys. Soc. Jpn*. **46**, 102–105 (1979); ibid, **46**, 11522 (1979).

[46] Sheldrick GM (2008). A short history of SHELX. Acta Crystallogr A.

